# Comprehensive Landscape of Ovarian Cancer Immune Microenvironment Based on Integrated Multi-Omics Analysis

**DOI:** 10.3389/fonc.2021.685065

**Published:** 2021-06-17

**Authors:** Jiacheng Shen, Tingwei Liu, Qiaoli Bei, Shaohua Xu

**Affiliations:** Department of Gynecology, Shanghai First Maternity and Infant Hospital, School of Life Sciences and Technology, Tongji University, Shanghai, China

**Keywords:** ovarian cancer, multi-omics, immune microenvironment, immune classification, immunotherapy

## Abstract

Epithelial ovarian cancer has a low response rate to immunotherapy and a complex immune microenvironment that regulates its treatment outcomes. Understanding the immune microenvironment and its molecular basis is of great clinical significance in the effort to improve immunotherapy response and outcomes. To determine the characteristics of the immune microenvironment in ovarian cancer, we stratified ovarian cancer patients into three immune subtypes (C1, C2, and C3) using immune-related genes based on gene expression data from The Cancer Genome Atlas and found that these three subtypes had significant differences in immune characteristics and prognosis. Methylation and copy number variant analysis showed that the immune checkpoint genes that influenced immune response were significantly hypermethylated and highly deleted in the immunosuppressive C3 subtype, suggesting that epigenetic therapy may be able to reverse the efficacy of immunotherapy. In addition, the mutation frequencies of BRCA2 and CDK12 were significantly higher in the C2 subtype than in the other two subtypes, suggesting that mutation of DNA repair-related genes significantly affects the prognosis of ovarian cancer patients. Our study further elucidated the molecular characteristics of the immune microenvironment of ovarian cancer, which providing an effective hierarchical method for the immunotherapy of ovarian cancer patients, and has clinical relevance to the design of new immunotherapies and a reasonable combination strategies.

## Introduction

Immunotherapy is an innovative treatment for cancer that has recently received a great deal of attention and has shown significant benefits in many types of cancer, but not ovarian cancer (OC) (1, 2). Recent studies show that the monotherapy used in many solid cancers has not led to substantial progress in the treatment of OC, with clinical response rates to single-dose immunotherapy of about 10-15% in advanced OC (3–5). Positive responses to immunotherapy often rely on interactions of tumor cells with immune regulation within the tumor microenvironment (TME). The TME has an essential role in suppressing or enhancing the immune response (6). Recent studies have shown that an immunosuppressive TME is a major obstacle to successful implementation of tumor immunotherapy in OC patients (7, 8). Therefore, understanding how the immune microenvironment of OC may hinder effective immune attack could guide the prediction of OC patients and promote more practical applications of immunotherapy in OC.

The tumor immune microenvironment (TIME) is highly complex. The composition of immune cells in the TIME may vary significantly among different patients with the same cancer type, suggesting that mapping the composition of immune infiltrates in the TIME and their functional status is essential for both diagnosis and treatment strategy design (9–11). Several studies have shown the prognosis of cancer patients could be inferred by immunotyping and that patient stratification based on immune genes is a feasible way to guide clinical treatment, suggesting that the establishment of immune subtypes could led to deeper understanding of the mechanism of tumorigenesis and provide a basis for precise clinical treatment (12, 13). A previous study based on transcriptome levels from The Cancer Genome Atlas (TCGA) identified four molecular subtypes (immunoreactive, mesenchymal, proliferative, and differentiated) of OC (14). However, the role of the immune microenvironment in the survival of patients has not been explored, and the differences in TIME among OC patients with different prognosis are not fully understood. Although several studies have identified various immune-related indicators that can predict the prognosis of OC patients, indicating that immune status affects prognosis (15–17), little is known about the molecular regulation that underlies different immune microenvironments.

In this study, gene expression profiles and immune-related gene (IRG) sets of 362 OC samples were obtained from TCGA and the ImmPort database, respectively. Non-negative matrix factorization (NMF) was used to analyze the expression profiles of IRGs in OC, and three immune-related molecular subtypes with distinct characteristics were identified. The immune characteristics, genomic characteristics, and clinical characteristics of the three subtypes were systematically analyzed, and the molecular basis of the immunosuppressive microenvironment of OC was characterized in detail using a combination of multi-omics analyses, including methylation and genome variation, to provide a new perspective for improving immunotherapeutic response in OC.

## Method and Materials

### Data Extraction

#### TCGA-OV Dataset

Gene expression profile data of OC patients were obtained from TCGA (https://tcga-data.nci.nih.gov/tcga/); the data type was RNA sequencing. The GDC API was used to download the latest OC clinical follow-up information from TCGA on 2019.09.14. Samples with missing clinical data or overall survival (OS) < 30 days, data for normal tissue samples, and genes with transcripts per kilobase million <1 in half of the samples were eliminated.

#### GEO Dataset

The GSE26712 and GSE153943 expression datasets were downloaded from NCBI (https://www.ncbi.nlm.nih.gov/geo/). Data of normal (non-tumor) tissue samples, samples with a survival time of less than 30 days or uncertain survival status were removed. The “bioconductor” package was used to map the microarray probe onto human gene symbol.

The statistical information of the preprocessed dataset is shown in **Table S1**.

#### Data of IRGs

IRGs were downloaded from the ImmPort database (https://immport.niaid.nih.gov), and duplicates were removed. Gene sets associated with 13 immune cell types were obtained from a previous study (18).

#### DNA Methylation and Genomic Variation Data

Methylation data (Illumina Human Methylation 27) of ovarian cancer patients were obtained from the UCSC database (https://gdc.xenahubs.net). The copy number variants (CNV) data of ovarian cancer patients was downloaded from the TCGA database using the ‘TCGABIOLinks’ package, and the data type was Masked Number Segment. SNP6 GRCh38 Remapped Probeset File (https://gdc.cancer.gov/about-data/gdc-data-processing/gdc-reference-files) was used as the markers file, and the CNV interval was mapped to the corresponding gene. Somatic single nucleotide variant (SSNV) data was the Mutect2 version in the TCGA database (https://www.cancer.gov/about-nci/organization/ccg/research/structural-genomics/tcga), which was the whole-exome sequencing data.

### Identification of Immune Subtypes in OC

The expression values of IRGs were extracted from the expression profile data of TCGA-OV, and genes with no or low expression were excluded. The genes in the top 50% with respect to the median absolute deviation (MAD) were screened for Cox univariate regression analysis to obtain significant prognosis-related genes. The NMF algorithm was used to construct a consistency matrix for the screened genes, and the samples were clustered. The optimal number of clusters was selected according to the three indicators: cophenetic (the larger the value, the more stable the clustering), dispersion, and rss (residual sum of squares; the smaller the value, the better the clustering effect of the model). We used the “sigclust” package and principal components analysis to compare the significance of clustering differences among subtypes. Furthermore, the random forest algorithm (“randomforest” package) was used to screen the most representative genes in each gene set.

### Relationship of Immune Subtypes with Clinical Characteristics

To determine the relationships between immune subtypes and clinical phenotypes, we analyzed the relationship between each subtype and age, stage, and grade, and observed the distribution of each subtype.

### Molecular Characteristics of Immune Subtypes

To observe each subtype’s function, we first analyzed the differences in gene expression among the three subtypes on a genome-wide basis. Differentially expressed genes (DEGs) in each group were screened using the “limma” package, and the hub genes in each subgroup were selected. Expression of genes related to epithelial-mesenchymal transition (EMT) and embryonic stem cell (ESC)-like genes is associated with malignancy and metastasis of human tumors. Therefore, we further analyzed the expression distribution of EMT pathway-related marker genes and ESC marker genes in the three subtypes.

### Immune Characteristics Analysis of Immune Subtypes

We used the “ESTIMATE” package to calculate ESTIMATEScore, ImmuneScore, and StromalScore values in each subtype, and analyzed the distribution differences of scores in the three subtypes. We further used the “MCPcounter” package to calculate the distribution differences of the ten immune components in each subtype, and used TIMER (https://cistrome.shinyapps.io/timer/) to calculate the scores of six types of immune cells in each sample for validation. In addition, we used metagene data from a previous study to estimate scores for 13 immune types and analyzed the differences in expression of eight common immunoassay genes among the three subtypes. We used the single-sample GSEA (ssGSEA) method in the “GSVA” package to calculate the scores of four immune pathways (IMMUNE_RESPONSE, IMMUNE_SYSTEM_PROCESS, IMMUNE_EFFECTOR_PROCESS, IMMUNE_SYSTEM_DEVELOPMENT) in each sample to analyze the enrichment differences among these immune pathways in each subtype.

### Analysis of Differentially Methylated Genes and Genomic Variation

The genes differentially methylated among subtypes were screened using the “limma” package with the selection criteria |log(fold change)| >0.3 and false discovery rate (FDR) <0.05. The “Hmisc” package was used to calculate the correlation between the transcription-level expression and the degree of methylation of each gene. If the correlation was negative, the expression level was considered to be related to the degree of methylation. Somatic variation data were retained in the mutation note format. Differentially mutated genes were identified by the “maftools” package with p<0.05 as the significance threshold. The online tool GISTIC2.0 (https://cloud.genepattern.org/) was used to analyze CNV data. Specific amplifications and deletions of chromatin sites in each immune subtype were then selected for subsequent analysis.

### Functional Enrichment Analysis

Genetic ontology (GO) and Kyoto Encyclopedia of Genes and Genomes (KEGG) pathway enrichment analysis were conducted using the “clusterprofiler” package, with FDR < 0.05 representing statistical significance.

### Cell Culture

Human OC cell lines SKOV3, A2780, HEY, and HO8910 were obtained from the Obstetrics and Gynecology Hospital Affiliated to Fudan University and grown at 37°C and 5% CO_2_ in RPMI-1640 (Servicebio, Wuhan, China) medium with UltraGRO™ cell culture supplement (AventaCell, Atlanta, USA) and penicillin-streptomycin (Yuchunbio, Shanghai, China). For 5-Azacytidine (5-AzaC) treatment, the growth medium was supplemented with 10 μmol/L or 100μmol/L 5-AzaC (MCE, USA) for 12 h or 24 h.

### Real-time PCR (RT‐PCR)

Total RNA from cells was extracted with TRIzol (Invitrogen, USA). After determination of its purity and concentration, RNA was reverse transcribed into cDNA using a 5X ALL-IN-One RT MasterMix kit (Applied Biological Materials Inc, Canada). RT-PCR was performed using a TB Green Premix Ex Taq kit (Takara, Japan). GAPDH was used as the internal control for all PCR reactions. The specific primers are listed in **Table S6**.

### Statistical Analysis

Univariate survival analysis was performed using the Cox risk regression model, and a significance threshold of log-rank p<0.05 was set to screen IRGs that were significantly associated with prognosis. Chi-square test was used to determine the significance of bias in the distribution of clinical characteristics among subtypes. Wilcoxon rank test was used to determine significance in comparisons of two groups of continuous variables, Kruskal-Wallis rank test was used for comparisons of more than two groups, and the Benjamini-Hochberg method was used to control the FDR. All the above analyses were performed using R version 3.5.1. Unless otherwise specified, ****P < 0.0001, ***P < 0.001, **P < 0.01, and *P < 0.05.

## Results

### Identification of Immune Subtypes in Ovarian Cancer

**Figure 1:**
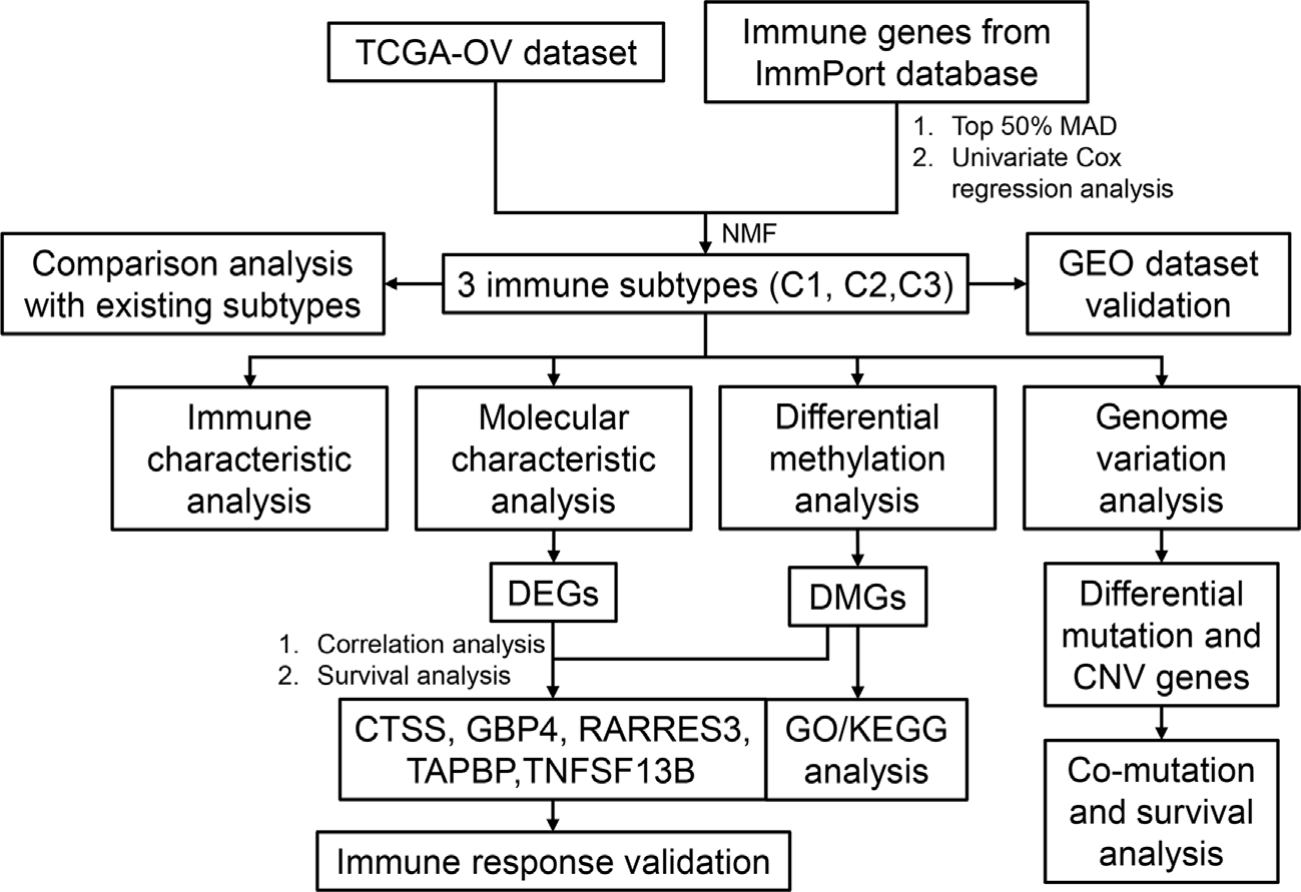
The workflow of study design.

**Figure 2:**
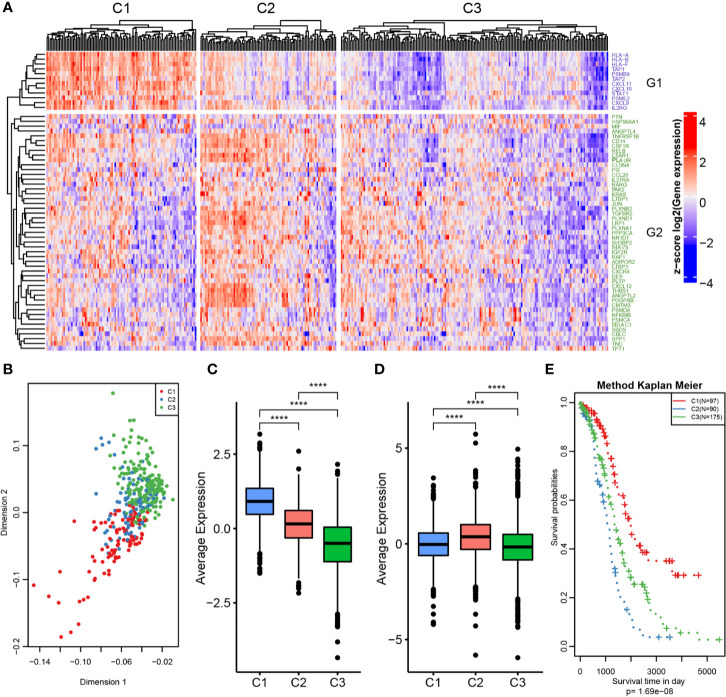
OC immunotyping based on IRGs. **(A)** Gene expression heatmap of OC immune subtypes. **(B)** Principal component analysis of the differences between immune subtypes. **(C)** The expression difference distribution box plot of the G1 gene set between subtypes was analyzed. **(D)** The expression difference distribution box plot of the G2 gene set between subtypes was analyzed. **(E)** The K-M curve of overall survival rate of immune subtypes in TCGA dataset was tested by log rank test. ****P < 0.0001.

The workflow of this study is shown in **Figure 1**. The expression values of IRGs were extracted from the TCGA dataset, and 798 IRGs were screened after removing genes with no or low expression. Furthermore, the genes whose MAD was in the top 50% were screened out for univariate Cox regression analysis, and 61 genes that were significantly related to prognosis were obtained **(** **Tables S2, S3** **)**. Using these prognosis-related genes, the optimal number of clusters obtained by the NMF algorithm was 3 **(** **Figures S1A, B** **)**; the three resulting clusters were defined as C1 (n=97), C2 (n=90), and C3 (n=175), respectively **(** **Figure 2A** **)**. The expression levels of prognosis-related immune genes were the highest in C1 and C2 but lowest in C3. Principal components analysis was used to analyze the expression profiles of these prognostic immune genes, revealing significant clustering differences between the three subtypes with different distribution characteristics. These results indicate that samples in the three clusters had significantly different molecular characteristics **(** **Figure 2B** and **Table S4** **)**. Moreover, there were significant differences in prognosis among the three immune subtypes, with C1 being the best and C2 the worst **(** **Figures 2E** and **S1D** **)**. This suggests that there are multiple groups of immune molecular subtypes in OC with significant differences in prognosis among them.

**Figure 3:**
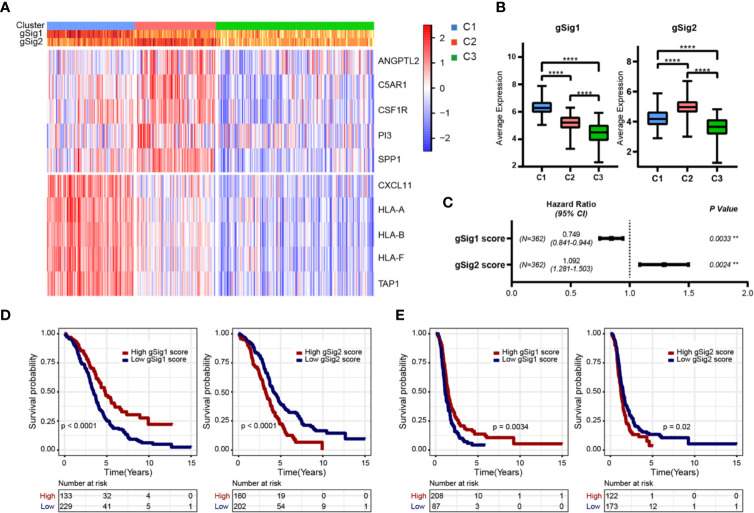
Selection of representative genes. **(A)** The expression heatmap of representative genes in two gene sets. **(B)** The expression difference distribution box plot of the gSig1 and gSig2 gene sets between subtypes was analyzed. **(C)** The prognosis relationship between the two gene sets and OS. **(D)** Relationship between two gene sets and OS in patients with ovarian cancer. **(E)** Relationship between two gene sets and PFS in patients with ovarian cancer. **P < 0.01, and ****P < 0.0001.

The 61 prognosis-related immune genes were divided into two categories: G1 and G2. The genes in G1 were highly expressed in C1 samples, whereas those in G2 were highly expressed in C2 samples **(** **Figures 2C, D** **)**. To further screen out representative prognostic genes, we selected the top 10 genes using the random forest algorithm, resulting in five G1 immune genes and five G2 immune genes **(** **Figures 3A** and **S1C** **)**. The gsig score of each immune subtype was expressed as the average expression level of the representative genes. Gsig1 was highly expressed in the C1 subgroup, whereas gsig2 was highly expressed in the C2 subgroup **(** **Figure 3B** **)**. Univariate analysis and survival analysis showed that gsig1 was a protective factor for OS and improved progression-free survival (PFS) in patients with high expression of G1 genes, with the opposite results for gsig2 **(** **Figures 3C–E** **)**.

### Relationship of Immune Subtypes With Clinical Characteristics

We analyzed the relationships between the three subtypes and age, grade and stage, respectively. As shown in **Table S5**, there was no significant relationship between any of the three subtypes and stage, age, or grade. This suggests that the three subtypes are independent of clinical characteristics.

### Relationship of Immune Subtypes With Molecular Characteristics

**Figure 4:**
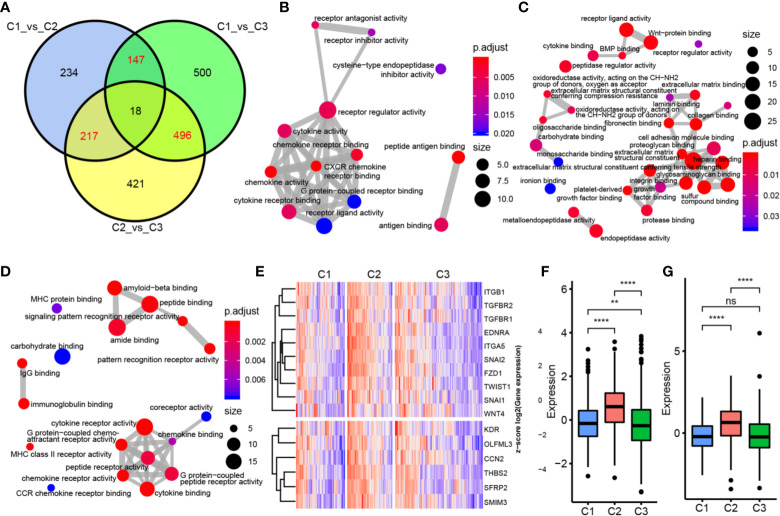
Functional analysis of immune gene module. **(A)** Wayne map of the difference genes in three subtypes. **(B)** GO pathway enriched by C1 subtype specific genes. **(C)** GO pathway enriched by C2 subtype specific genes. **(D)** GO pathway enriched by C3 subtype specific genes. The thickness of the line indicates the number of shared genes; the color indicates FDR, and the node size indicates the number of enriched genes. **(E)** The upper panel represents the expression profile clustering heat map of EMT marker genes, and the lower panel represents the expression profile clustering heat map of ESC marker genes. **(F)** Box plot of expression and distribution of EMT marker gene in three subtypes. **(G)** The expression distribution of ESC marker gene in three subtypes was different by box line diagram. **P < 0.01, ****P < 0.0001, and ns, ‘No Significant’.

To determine the function of each subtype, we first analyzed the differences in gene expression among the three subtypes based on the whole genome. There were 616 DEGs between C1 and C2, 1161 DEGs between C1 and C3, and 1152 DEGs between C2 and C3 **(** **Figure 4A** **)**. We then selected genes (147 in C1, 217 in C2, and 496 in C3) with specific differences in each subtype for GO and KEGG enrichment analyses. C1-specific genes were enriched in 13 biological pathways, which were mainly related to receptor regulator activity, cytokine receptor binding, and cytokine activity. C2 was enriched in 38 biological pathways, mainly related to growth factor binding and Wnt-protein binding. C3 was enriched in 19 biological pathways, mainly related to MHC class II receptor activity and G protein-coupled peptide receptor activity **(** **Figures 4B–D** **)**. We also identified EMT pathway-related marker genes and ESC marker genes and analyzed their expression distributions in the three subtypes. The results showed that most of these genes had significantly higher expression in C2 compared with C3 and C1, indicating that the C2 subtype may be more aggressive and malignant than the others **(** **Figures 4E–G** **)**.

### Immune Characteristics Analysis of Immune Subtypes

**Figure 5:**
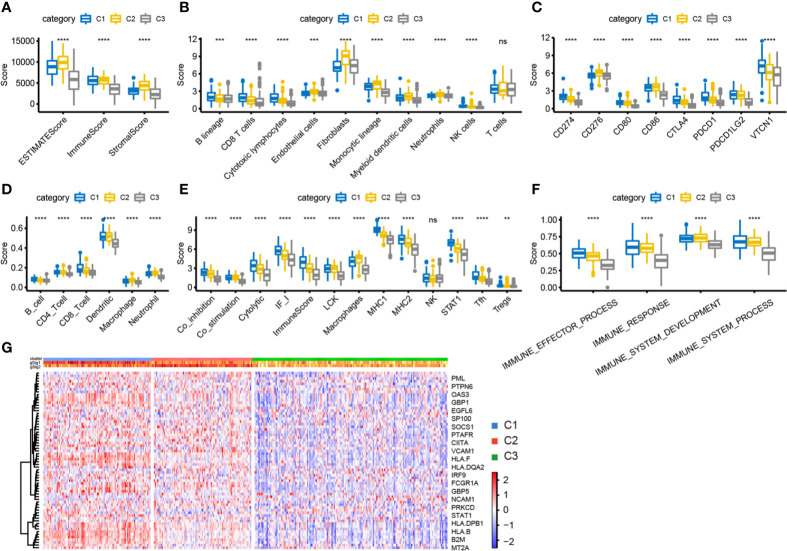
Analysis of immune characteristics of immune subtypes. **(A)** The box plot of immune score difference in each subtype was calculated by ESTIMATE. **(B)** The box plot of 10 immune components difference in each subtype was calculated by MCPcounter. **(C)** The expression differences of 8 common immune checkpoint genes among three subtypes. **(D)** The difference of 6 kinds of immune cell scores in each subtype. **(E)** The differences of 13 kinds of immune components in different subtypes. **(F)** Differences of enrichment scores of four immune pathways among three subtypes. **(G)** Differential expression of IFN-γ related regulatory genes in three immune subtypes. **P < 0.01, ****P < 0.0001, and ns, ‘No Significant’.

We used the “ESTIMATE” package to calculate immune scores for each subtype and found that the immune score and stromal score were highest in C2 and lowest in C1 **(** **Figure 5A** **)**. We further calculated the distribution differences of 10 immune components using the “MCPcounter” package. The results showed significantly higher abundance of fibroblasts and cells of monocytic lineage in C2, and of CD8^+^ T cells and cells of B lineage in C1, whereas the abundance of T cells in C2 was significantly lower than that in C1 and C3 **(** **Figure 5B** **)**. Similar results were obtained by using TIMER to calculate the scores of six types of immune cells in each sample **(** **Figure 5D** **)**. Then, we estimated the scores of 13 immune cell types using metagene data collected in a previous study; again, their scores were significantly higher in C1 than in C2 and C3 **(** **Figure 5E** **)**. These results indicate that the C1 subtype tumor microenvironment has a higher content of immune cells than that of C2 and C3, with more immune activity. We analyzed the expression of eight common immune checkpoint genes in the three subtypes and found that five genes had higher expression in C1 subtype was higher than in C2, CD276 and CD86 had significantly higher expression in C2 subtype than in the other two groups, and PDCD1LG2 had higher expression in C1 and C2 subtypes than in C3. These results indicate that the three subtypes might have different clinical responses to immunotherapy **(** **Figure 5C** **)**. We also used the ssGSEA method in the “GSVA” package to calculate the enrichment scores of four immune pathways (IMMUNE_RESPONSE, IMMUNE_SYSTEM_PROCESS, IMMUNE_EFFECTOR_PROCESS, and IMMUNE_ SYSTEM_DEVELOPMENT) in each subtype. The scores were significantly lower in the C3 subtype compared with the C1 and C2 subtypes **(** **Figure 5F** **)**. Moreover, the expression of IFN-γ pathway-related genes was significantly lower in the C3 subtype than in the other two subtypes **(** **Figure 5G** **)**. In summary, immune-enhanced subtypes C1 and C2 showed significant differences in various immune characteristics, which led to opposite clinical results.

### External Dataset Validation for Immunotyping

**Figure 6:**
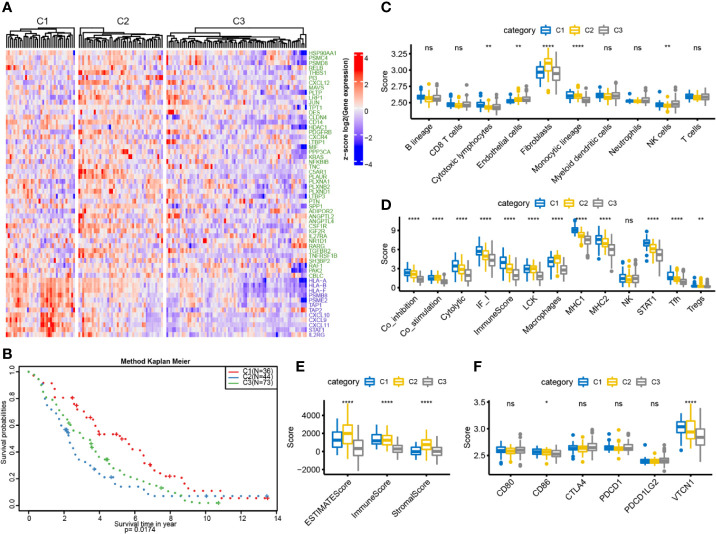
OC immunotyping based on IRGs in validation set. **(A)** Gene expression heatmap of OC immune subtypes. **(B)** The K-M curve of the overall survival rate of the immune subtypes in the validation dataset, measured by log rank test. **(C)** 10 immune components difference in each subtype. **(D)** The differences of 13 kinds of immune components in different subtypes. **(E)** The box plot of immune score difference in each subtype was calculated by ESTIMATE. **(F)** The expression differences of 6 common immune checkpoint genes among three subtypes. *P < 0.05, **P < 0.01, ****P < 0.0001, and ns ‘No Significant’.

To further verify characteristics of the three subtypes, we downloaded the GSE26712 standardized dataset from the GEO database. This comprised 195 samples, from which 153 samples with clinical follow-up information were selected, and the expression profiles of characteristic genes were extracted. The samples were classified by the training model, and 36 samples of C1 subtype, 44 samples of C2 subtype, and 73 samples of C3 subtype were predicted. The G1 gene set was highly expressed in C1, and the G2 gene set was highly expressed in C2 **(** **Figure 6A** **)**. Similarly, the differences in prognosis among the three groups were analyzed: C1 had significantly better prognosis than C3, and C3 was significantly better than C2, consistent with the results obtained in the training set **(** **Figure 6B** **)**. Furthermore, we used MCPcounter to analyze ten immune components and found significant differences among the three samples **(** **Figure 6C** **)**. We analyzed the expression distribution of 13 immune metagenes in each subtype and found that most of the metagenes were highly expressed in C1, consistent with the results in the validation set **(** **Figure 6D** **)**. Further analysis of the sample immune scores showed that these were significantly higher in the C2 group than in the other groups, consistent with the results from the training set **(** **Figure 6E** **)**. We analyzed the gene expression distribution of immune checkpoints; the results for two of the six genes were consistent with those obtained in the training set **(** **Figure 6F** **)**. The above results show that our immune subtype classification method can be verified in an external independent dataset and thus has portability.

### Comparison of Immune Subtypes and Existing Subtypes

**Figure 7:**
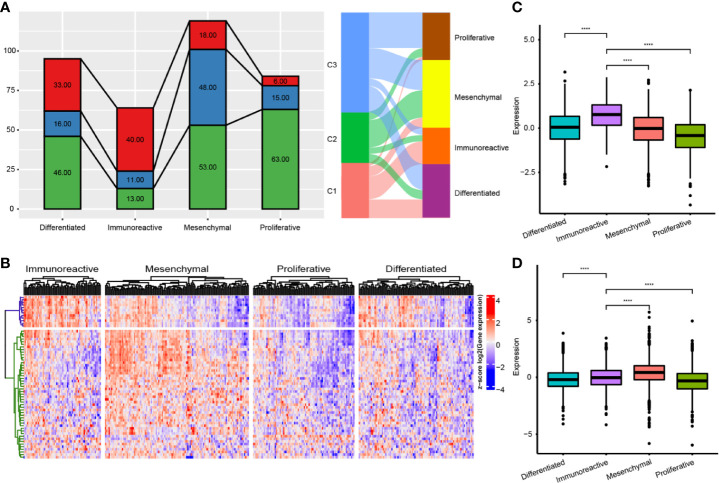
Comparison of immune subtypes and existing subtypes. **(A)** The intersection of molecular subtypes and immune subtypes in TCGA. **(B)** Clustering heatmap of prognosis related immune genes in four subtypes. **(C)** Differential distribution of G1 gene set expression among immune subtypes. **(D)** Differential distribution of G1 gene set expression among immune subtypes. ****P < 0.0001.

We compared the differences among the four previously reported molecular subtypes and our three immune subtypes, and found that the C1 subtype was significantly enriched in the immunoreactive subtype (P<0.001), the C2 subtype was significantly enriched in the mesenchymal subtype (P<0.001), and the C3 subtype was significantly enriched in the proliferative subtype (P<0.001) **(** **Figure 7A** **)**. We further analyzed the expression distribution of immune prognosis-related genes in four TCGA subtypes and found that their expression level in the immunoreactive and mesenchymal subtypes were significantly consistent with those in the C1 and C2 subtypes **(** **Figure 7B** **)**. The expression level of the G1 gene set was significantly higher in the immunoreactive subtype than in other subtypes, whereas the expression level of the G2 gene set was significantly higher in the mesenchymal subtype **(** **Figures 7C, D** **)**.

### Relationship of Immune Subtypes with Methylation Status

**Figure 8:**
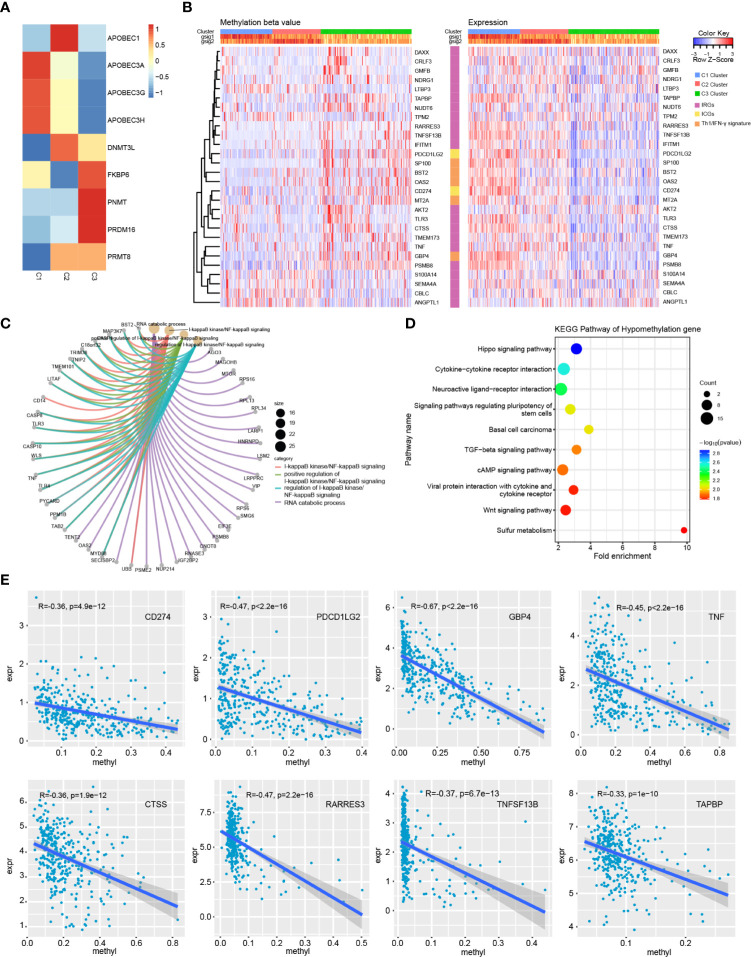
Methylation analysis of immune subtypes. **(A)** Expression differences of methylation related genes in three immune subtypes. **(B)** Heatmap of genes with significant differences in methylation and transcriptional expression among subtypes. **(C)** GO enrichment analysis of hypermethylated genes in C3 subtype. **(D)** KEGG pathway analysis of hypomethylated genes in C3 subtype. **(E)** Correlation trend of methylation level and transcription expression of immune related genes.

**Figure 9:**
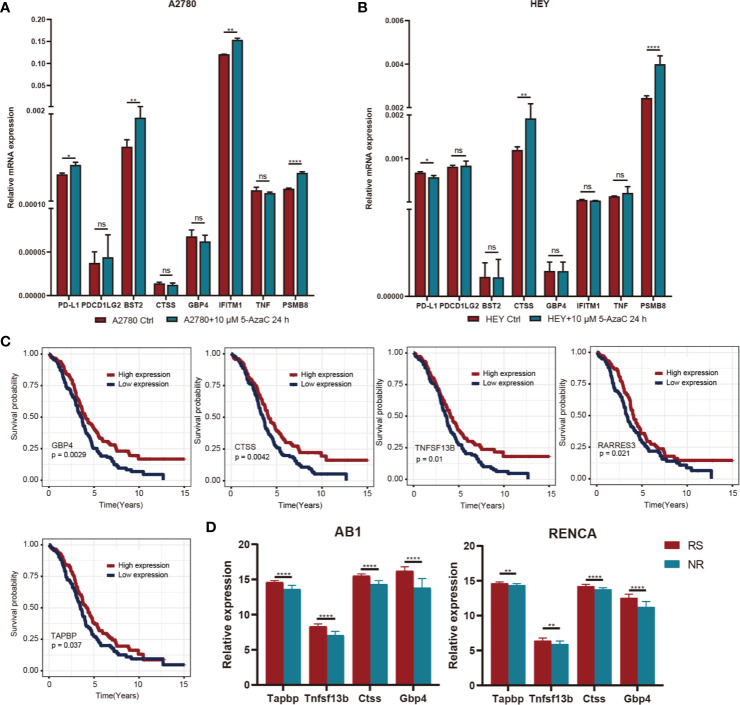
**(A)** After A2780 cell lines were treated with 5-AZ at the concentrations of 10μM for 24 hours, the changes of gene expression levels were observed. **(B)** After HEY cell lines were treated with 5-AZ at the concentrations of 10μM for 24 hours, the changes of gene expression levels were observed. **(C)** K-M survival curve of differentially methylated genes associated with prognosis. **(D)** Relationship between prognosis related differential methylation genes and immunotherapy responsiveness. *P < 0.05, **P < 0.01, ****P < 0.0001, and ns, ‘No Significant’.

To explore the underlying causes of low immunoreactivity in patients with the C3 subtype, we further analyzed the degree of gene methylation in patients with OC. First, we determined the expression levels of methylation- and demethylation-related genes in the three immune subtypes, and found decreased expression levels of demethylation genes in C3. By contrast, the expression levels of methylation genes increased significantly in C3, indicating that the gene methylation status of the C3 subtype was different from that of C1 and C2, and that the immune reactivity of this subtype might be related to the degree of methylation **(** **Figure 8A** **)**. We further selected the 27K methylation dataset from TCGA to screen differential methylation genes among the three immune subtypes. The methylation levels of many IRGs (including CD274 and PDCD1LG2) and IFNγ-related genes were significantly increased in C3 compared with C1 and C2, whereas their expression levels were significantly decreased **(** **Figures 8B, E** **)**. In the C3 subtype, hypermethylated genes were significantly enriched in the NF-κB pathway, whereas hypomethylated genes were mainly enriched in cytokine receptor interaction and the TGF-β signaling pathway **(** **Figures 8C, D** **)**. Moreover, there was no significant correlation between methylation status and clinical information of OC patients, indicating that our immune subtype classification method was better able to classify the immune response types of patients compared with clinical data. To verify the effects of methylation status on these IRGs, we treated OC cells with DNA methylation inhibitor 5-AzaC. The results showed that the expression levels of some genes increased after DNA methylation was inhibited **(** **Figures 9A, B** and **S2** **)**. In conclusion, the low immune reactivity and prognosis of C3 subtype patients are related to the methylation of IRGs, such as CD274. We screened the genes with different methylation levels and transcriptional expression, and obtained five genes related to the prognosis of OC patients. By verifying the immune response of tumor patients in a GEO data sets (GSE153943), we found that these genes had significantly differential expression in patients with different immune responses **(** **Figures 9C, D** **)**.

### Relationship of Immune Subtypes and Genomic Variation

**Figure 10:**
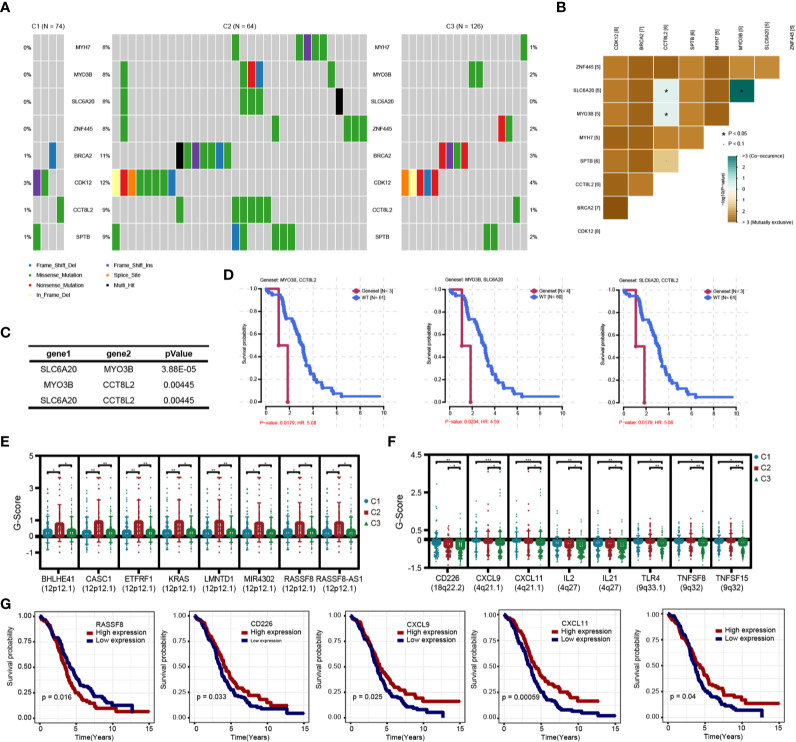
Genomic variation analysis of immune subtypes. **(A)** Differential high frequency mutation genes in C2 subtypes. **(B)** Correlation heatmap of co-mutated genes in C2 subtype. **(C)** Correlation of co-mutated genes in C2 subtypes. **(D)** Survival and prognosis of patients with co-mutated genes. **(E)** Significantly increased copy number genes in C2 subtype. **(F)** Significantly decreased copy number genes in C3 subtype. **(G)** Survival curve of differential copy number genes associated with prognosis.

We analyzed the gene mutations among the three immune subtypes **(** **Figures S3A–C** **)** and found eight genes with significant mutation differences in the C2 subtype compared with the other two subtypes, including BRCA2 and CDK12 **(** **Figure 10A** **)**; moreover, the proportions of these eight genes in C2 were significantly higher than those in C1 and C3 **(** **Table S7** **)**. We further analyzed the mutations of DNA damage repair genes related to BRCA1 and BRCA2, and found that the mutation frequency of BRCA1 (but not that of BRCA2) in the C2 subtype was slightly lower than that in the other two subtypes, but there was no significant statistical difference **(** **Figures S3D, E** **)**. Further analysis showed that CCT8L2, SLC6A20, and MYO3B had co-mutation tendencies in OC **(** **Figures 10B, C** **)**. In addition, among the eight genes with significant mutations in C2 subtype, co-mutations of MYO3B and CCT8L2, MYO3B and SLC6A20, or SLC6A20 and CCT8L2 were associated with significantly poorer prognosis **(** **Figure 10D** **)**. In order to further understand the impact of genomic variation on the three immune subtypes, we analyzed and screened the CNVs of each subtype. CASC1, KRAS, and RASSF8 showed significantly higher amplification in the C2 subtype compared with the C1 and C3 subtypes, whereas there was more deletion of various immune genes, including CD226, CXCL9, CXCL11, and IL2, in the C3 subtype **(** **Figures 10E–G** **)**. These results suggest that mutations of these genes or variations of genome copy number have an essential influence on prognosis in the C2 and C3 subtypes.

## Discussion

OC is a malignant tumor with a poor prognosis and the leading mortality rate among gynecological malignancies (19, 20). Despite significant advances in surgical treatments and chemotherapy in recent years, the survival rate for OC has improved only slightly (21, 22). Several experimental and clinical studies have shown that immunotherapy has incomparable advantages over traditional anti-tumor therapies in terms of extending PFS and OS, especially in patients with advanced cancer (23, 24). However, this response occurs only in a relatively small number of patients (25). Currently, single-dose immunotherapy in advanced OC has an unsatisfactory clinical response rate of about 10–15% (4, 5). Positive response to immunotherapy depends on immunomodulatory interactions within tumor cells and the TME, and the TME has a vital role in suppressing or enhancing the immune response (26). Therefore, a comprehensive understanding of the unique immune microenvironment of OC is of great significance to improve response to immunotherapy and patient outcomes.

Previous studies have shown that IRGs and the extent of immune cell infiltration can reflect tumor immunotherapy response (27). In this study, we identified three immune subtypes of OC based on 798 IRGs. These three immune subtypes have different immune microenvironments and molecular characteristics. C1 and C2 represent an immunologically activated state, whereas C3 represents an immunologically suppressed state, and the three subtypes were associated with significantly different survival rates. These results suggest that the immune characteristics of tumors determine the immune response rate and the effectiveness of immunotherapy. In combination with molecular typing of OC from TCGA, we further analyzed the uniqueness of immunotyping. We found that molecular typing was consistent with immunotyping, with significant enrichment of C1, C2, and C3 in immunoreactive, mesenchymal, and proliferative types, respectively. It is worth noting that nearly half of OC patients’ tumor microenvironments were in an immunosuppressive state, and the expression levels of immune checkpoint genes in these patients were significantly decreased. Although the expression of IRGs in the C2 subtype did not decrease significantly, patients with this subtype had the worst prognoses. These problems indicate that it is impossible to analyze the immune microenvironment of OC patients completely by transcriptional analysis alone. Recent studies have shown that the combination of multi-omics big data and bioinformatics analysis can provide a basis for an in-depth understanding of the mechanisms of tumorigenesis and guide precision clinical treatment (28, 29). Therefore, we further used multi-omics to further study the molecular characteristics of OC.

Usually, tumor-related molecular changes occur mainly in the genome, but evidence increasingly shows a critical role for epigenetics in the development and treatment of tumors (30). We found that several immune checkpoint genes, including those encoding PD-L1 (CD274) and PD-L2 (PDCD1LG2), showed significant hypermethylation in the C3 subtype, and the transcriptional expression level of these genes was significantly decreased, suggesting that the inadequate immune response of the C3 subtype may be related to the methylation status of immune checkpoint genes (31). Further functional enrichment analysis of differentially methylated genes showed significant associations with the NK-κB pathway, cytokine activity, and the TGF-β pathway, consistent with previous studies (32–34). We then identified five genes related to prognosis after screening genes with different methylation and transcriptional expression levels in the C3 subtype. The expression levels of four of these five genes (GBP4, CTSS, TNFSF13B, RARRES3, and TAPBP) were closely related to immunotherapy, according to validation in the GEO database and the results of previous studies (35–39). These results suggest that increasing the expression of immune checkpoint genes *via* epigenetic therapy can reverse the immunosuppressive microenvironment of OC and enhance the efficacy of immunotherapy (40, 41).

The C2 subtype had a significantly different mutation pattern compared with the other two subtypes, involving high-frequency mutations of BRCA2 (11%) and CDK12 (12%), especially missense mutations. In previous studies, these two genes have been proved to be associated with the tumor progression of OC and breast cancer, indicating that genomic mutations may drive the prognosis of patients with the C2 subtype; BRCA mutations may also affect the efficacy of PARP inhibitors in OC patients (42, 43). In addition, potential co-mutations were found among SLC6A20 (8%), MYO3B (8%), and CCT8L2 (9%), and patients with co-mutations in both genes had significantly worse prognosis than those without the mutations. In previous studies, these genes were shown to affect tumor progression *via* changes in expression at the transcriptional level; however, in this study, we studied their impact on prognosis at the level of genomic mutation (44–46). CNV analysis showed that genes related to chemokines, cytokines, and immune response receptors, including CD226, CXCL9, CXCL11, and IL2, were deleted to varying degrees in the C3 subtype. Moreover, the low expression of these genes was significantly related to poor prognosis in patients with OC, and we found that they had previously been validated as associated with tumor immune response and immunotherapeutic efficacy (47–49).

In summary, in this study, we used immune genes to classify OC patients and combined these results with multi-omics data to analyze the causes of the different immune microenvironment characteristics of different immune subtypes. We further revealed differences in immune response patterns and prognosis of OC patients at the molecular level. However, the results need to be further validated in clinical samples of patients receiving immunotherapy to clarify further the factors affecting immune response. Overall, our study provides a conceptual framework for understanding the TIME of OC and may have clinical significance for the design of novel immunotherapies and appropriate combination strategies.

## Data Availability Statement

The datasets presented in this study can be found in online repositories. The names of the repository/repositories and accession number(s) can be found in the article/**Supplementary Material**.

## Author Contributions

JS, TL, and QB collected and assembled the data. JS and TL performed data analysis and QB performed interpretation. JS, TL, and QB wrote the manuscript. SX provided conception and helped with manuscript and data review finally. All authors contributed to the article and approved the submitted version.

## Funding

This work was supported by the National Natural Science Foundation of China (grant no. 81772762 and 81502227), Clinical science and technology innovation project of Shanghai Shenkang Hospital Development Center (grant no. SHDC12019X34) and Natural Science Foundation of Shanghai (grant no. 21ZR1450900).

## Conflict of Interest

The authors declare that the research was conducted in the absence of any commercial or financial relationships that could be construed as a potential conflict of interest.

## Abbreviations

TCGA, The Cancer Genome Atlas; GEO, Gene Expression Omnibus; OC, Ovarian cancer; TME, Tumor microenvironment; TIME, Tumor immune microenvironment; IRG, Immune related gene; CNV, Copy number variants; MAD, Median absolute deviation; DEG, Differentially expressed gene; DMG, Differentially methylated gene; EMT, Epithelial-mesenchymal transition; ESC, Embryonic stem cells; FDR, False discovery rate; GO, Genetic ontology; KEGG, Kyoto encyclopedia of genes and genomes; PFS, Progression-free survival; OS, Overall survival.
