# Supplementary material for: Comprehensive Landscape of Ovarian Cancer Immune Microenvironment Based on Integrated Multi-Omics Analysis
Source: Front Oncol. 2021 Jun 17;11:685065. doi: 10.3389/fonc.2021.685065 (PMC8247482; doi:10.3389/fonc.2021.685065)
Supplement: Supplementary file 1 [file DataSheet_1.docx]

Supplementary Material

# Supplementary Material

**Support vector machine (SVM) is used to verify the immune classification method with external data in GEO dataset GSE26712. The verification process code is as follows:**

library(e1071)

tObj = tune.svm(scale(t(TCGA.sigGenes.exp)),factor(TCGA.cluster),

probability = TRUE,

cost=c(0.001,0.01,0.1,1,5,10,100,1000), scale=F)

geo.cluster = predict(tObj$best.model,scale(t(GEO.sigGenes.exp)))

# Supplementary Figures and Tables

## Supplementary Figures


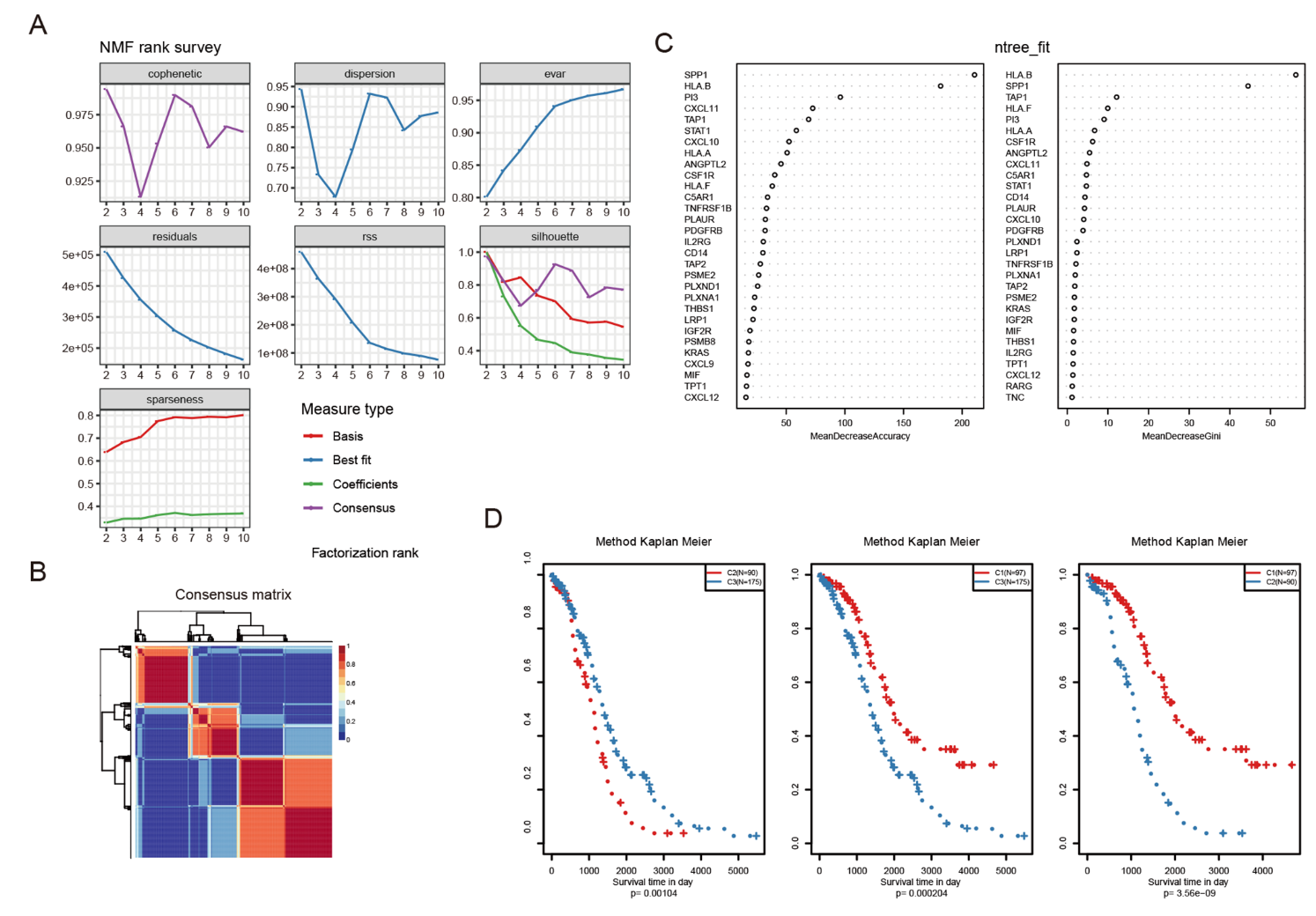


**Supplementary Figure 1.** **(A)** Parameters of NMF algorithm in selecting the best clustering. **(B)** NMF algorithm to determine the best clustering. **(C)** Selection of representative genes of two gene sets by random forest algorithm. **(D)** Survival differences among three subtypes.


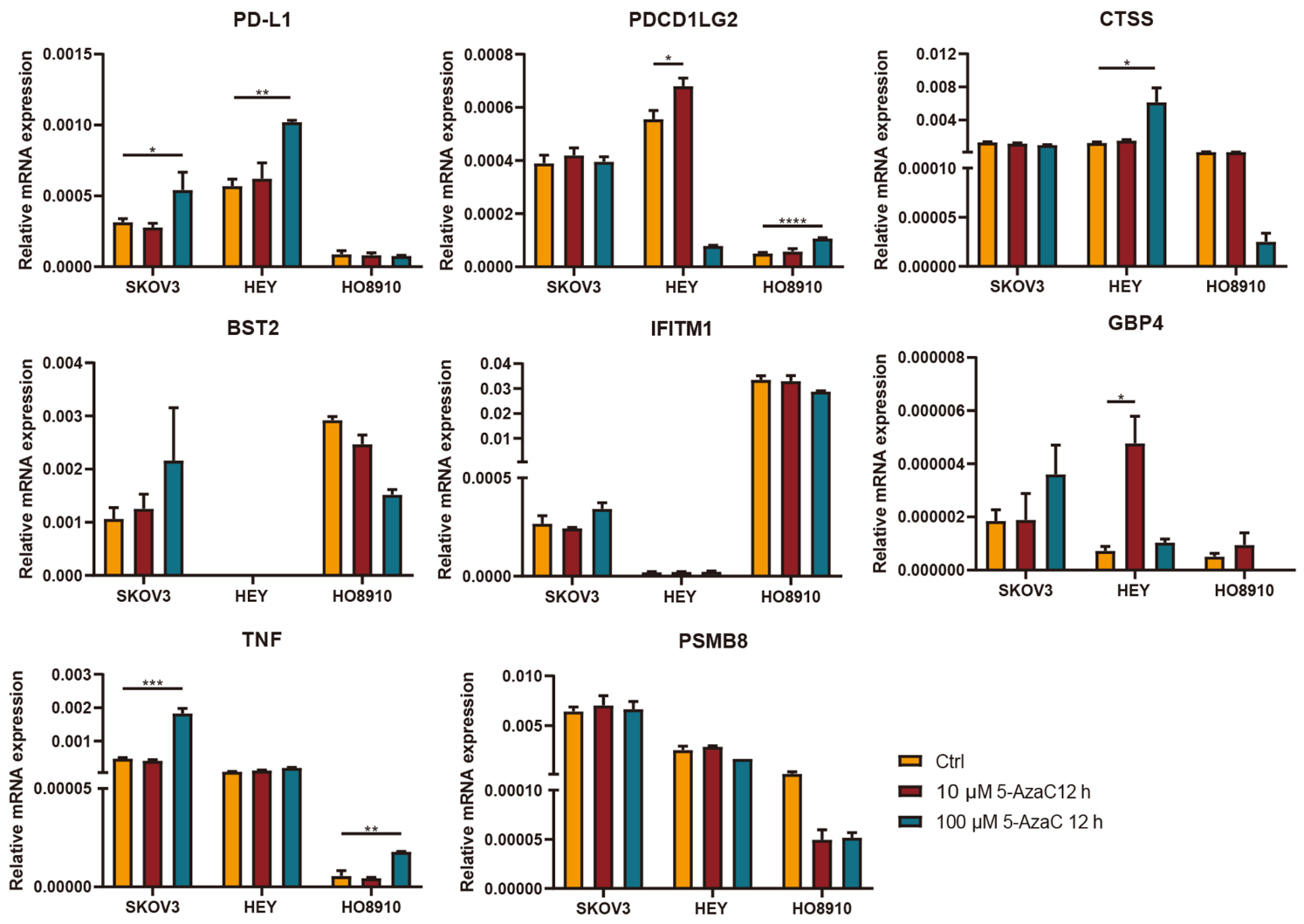


**Supplementary Figure 2.** After SKOV3, HEY and HO8910 cell lines were treated with 5-az at the concentrations of 10μM and 100μM for 12 hours, the changes of gene expression levels were observed.


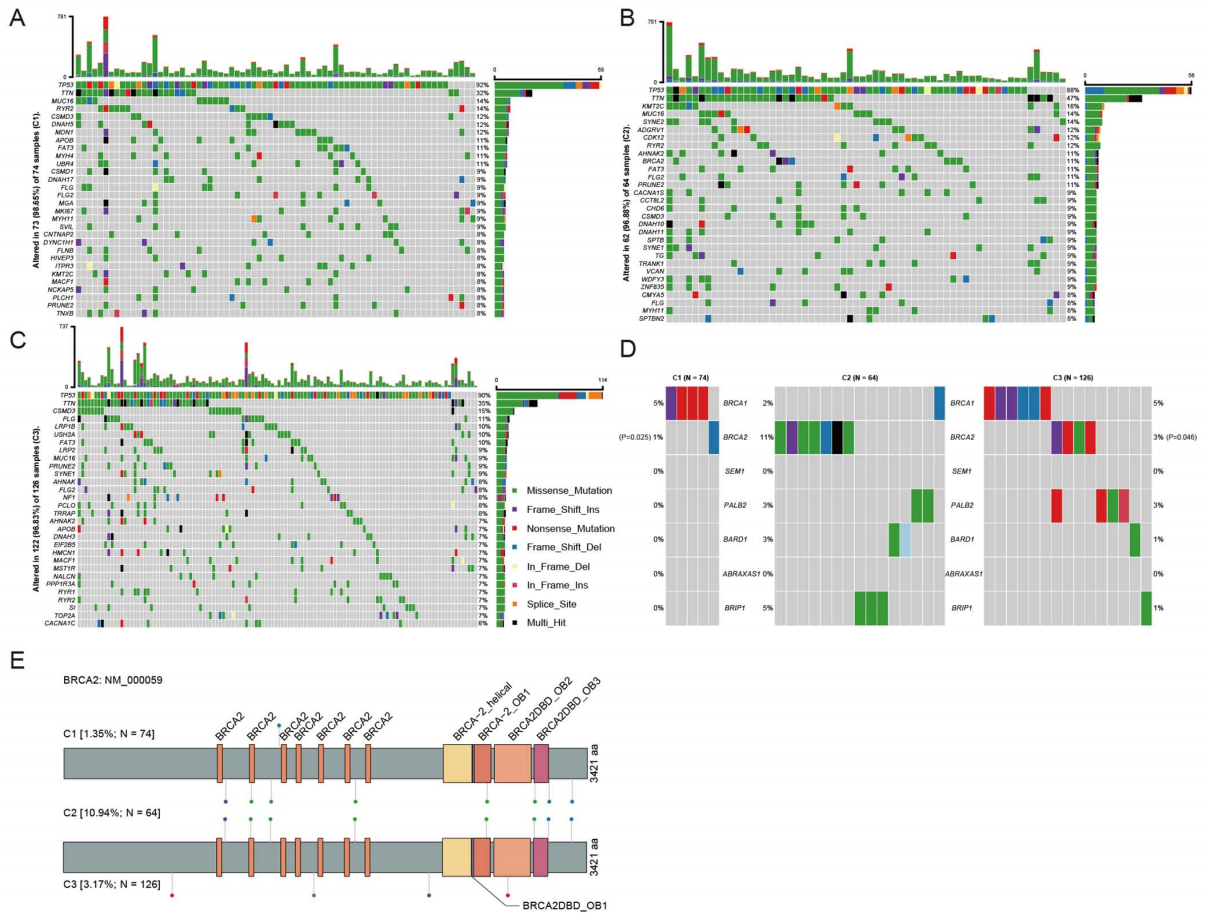


**Supplementary Figure 3.** **(A)-(C)** Mutation pattern of each subtype (displayed the top 30 genes). **(D)** The difference of mutation frequency of BRCA1 and BRCA2 related DNA damage repair genes among different subtypes. **(E)** The mutation details of differential mutation gene BRCA2 in the three subtypes.

## Supplementary Tables

**Table S1.** Clinical information of patients with OC in TCGA and GEO database after preprocess.

| **Features** | | **TCGA** | **GSE26712** |
| --- | --- | --- | --- |
| Sample number | | 362 | 153 |
| Stage (%) | Stage I | 1 ( 0.3) |  |
|  | Stage II | 20 ( 5.6) |  |
|  | Stage III | 284 (79.1) |  |
|  | Stage IV | 54 (15.0) |  |
| Grade (%) | G1 | 1 ( 0.3) |  |
|  | G2 | 42 (11.7) |  |
|  | G3 | 309 (85.8) |  |
|  | G4 | 1 ( 0.3) |  |
|  | GB | 1 ( 0.3) |  |
|  | GX | 6 ( 1.7) |  |
| OS Dead = 1 (%) | | 221 (61.0) | 129 (84.3) |
| Age (%) | <=60 | 199 (55.0) |  |
|  | >60 | 163 (45.0) |  |

**Table S2.** Prognostic genes for immunophenotyping.

| Gene | p-value | HR | Low 95%CI | High 95%CI |
| --- | --- | --- | --- | --- |
| HLA-A | 0.034981 | 0.999682 | 0.999387 | 0.999978 |
| HLA-B | 0.038665 | 0.999799 | 0.999608 | 0.99999 |
| HLA-F | 0.004917 | 0.988966 | 0.981349 | 0.996643 |
| HSP90AA1 | 0.044008 | 0.999446 | 0.998906 | 0.999985 |
| PSMB8 | 0.012621 | 0.997974 | 0.996385 | 0.999566 |
| PSMC4 | 0.023272 | 1.000815 | 1.000111 | 1.001519 |
| PSMD8 | 0.02992 | 1.000862 | 1.000084 | 1.001641 |
| PSME2 | 0.040155 | 0.997336 | 0.994798 | 0.99988 |
| RELB | 0.01316 | 1.008165 | 1.001705 | 1.014666 |
| TAP1 | 0.001494 | 0.995651 | 0.992976 | 0.998333 |
| TAP2 | 0.049561 | 0.986774 | 0.973749 | 0.999974 |
| THBS1 | 0.011432 | 1.002266 | 1.00051 | 1.004025 |
| PI3 | 0.002879 | 1.000413 | 1.000141 | 1.000685 |
| CXCL10 | 0.005662 | 0.998927 | 0.998168 | 0.999687 |
| CXCL9 | 0.002716 | 0.994818 | 0.991445 | 0.998202 |
| CXCL11 | 0.000668 | 0.990173 | 0.984556 | 0.995822 |
| CXCL12 | 0.000206 | 1.006124 | 1.002886 | 1.009373 |
| MAVS | 0.039162 | 1.008109 | 1.000401 | 1.015875 |
| PLTP | 0.018331 | 1.000625 | 1.000106 | 1.001144 |
| LRP1 | 0.000741 | 1.005957 | 1.002492 | 1.009434 |
| STAT1 | 0.021998 | 0.998114 | 0.996502 | 0.999728 |
| JUN | 0.024368 | 1.000734 | 1.000095 | 1.001373 |
| TPT1 | 0.005257 | 1.000329 | 1.000098 | 1.000561 |
| DES | 0.006737 | 1.004565 | 1.001261 | 1.007881 |
| CLDN4 | 0.014598 | 1.000932 | 1.000184 | 1.001681 |
| CCL28 | 0.008166 | 1.006378 | 1.001648 | 1.01113 |
| CD14 | 0.025808 | 1.001149 | 1.000139 | 1.00216 |
| HDAC1 | 0.03787 | 1.003474 | 1.000194 | 1.006764 |
| PDGFRB | 0.026762 | 1.005045 | 1.000579 | 1.00953 |
| CXCR4 | 0.035619 | 0.998718 | 0.997523 | 0.999914 |
| LTBP1 | 0.034909 | 1.004445 | 1.000314 | 1.008592 |
| MIF | 0.035666 | 0.999712 | 0.999443 | 0.999981 |
| PPP3CA | 0.004109 | 1.015209 | 1.004797 | 1.02573 |
| KRAS | 0.016858 | 1.001578 | 1.000284 | 1.002874 |
| NFKBIB | 0.038162 | 1.004944 | 1.000269 | 1.00964 |
| SBDS | 0.020212 | 1.006333 | 1.000986 | 1.01171 |
| TNC | 0.006971 | 1.006493 | 1.001772 | 1.011236 |
| C5AR1 | 0.000262 | 1.015753 | 1.007263 | 1.024315 |
| PLAUR | 0.019285 | 1.008586 | 1.001389 | 1.015833 |
| PLXNA1 | 0.00213 | 1.009855 | 1.003555 | 1.016195 |
| PLXNB2 | 0.011824 | 1.001575 | 1.000348 | 1.002803 |
| PLXND1 | 0.012188 | 1.006873 | 1.001495 | 1.01228 |
| CMTM3 | 0.021776 | 1.006362 | 1.000924 | 1.011828 |
| LTBP3 | 0.033662 | 1.002961 | 1.000228 | 1.005701 |
| PTN | 0.039131 | 0.998416 | 0.996913 | 0.999921 |
| SPP1 | 0.012623 | 1.000299 | 1.000064 | 1.000534 |
| ADIPOR2 | 0.01374 | 1.01315 | 1.002676 | 1.023733 |
| ANGPTL2 | 0.022686 | 1.0056 | 1.000781 | 1.010442 |
| ANGPTL4 | 0.000781 | 1.008108 | 1.00337 | 1.012869 |
| CSF1R | 0.01465 | 1.004106 | 1.000808 | 1.007415 |
| IGF2R | 0.017492 | 1.011732 | 1.002045 | 1.021513 |
| IL27RA | 1.00E-04 | 1.008296 | 1.004108 | 1.012501 |
| IL2RG | 0.032582 | 0.991285 | 0.983359 | 0.999274 |
| NR1D1 | 0.033353 | 1.009178 | 1.000721 | 1.017707 |
| RARG | 0.019932 | 1.005225 | 1.000824 | 1.009646 |
| TGFBR2 | 0.010599 | 1.006472 | 1.001505 | 1.011464 |
| TNFRSF1B | 0.035283 | 1.008825 | 1.000606 | 1.017111 |
| SH3BP2 | 0.037233 | 1.01133 | 1.000667 | 1.022106 |
| RAF1 | 0.038639 | 1.010173 | 1.000529 | 1.01991 |
| PAK2 | 0.046623 | 1.005278 | 1.000079 | 1.010505 |
| CBLC | 0.027873 | 1.003341 | 1.000363 | 1.006328 |

**Table S4.** Significance analysis of cluster differences among three subtypes.

|  | **C1** | **C2** | **C3** |
| --- | --- | --- | --- |
| **C1** | 1 | 1.49E-54 | 1.82E-96 |
| **C2** | 1.49E-54 | 1 | 2.18E-294 |
| **C3** | 1.82E-96 | 2.18E-294 | 1 |

**Table S5.** Relationship between three subtypes and clinical features.

| **Features** | **C1** | **C2** | **C3** | **p** |
| --- | --- | --- | --- | --- |
| Sample number | 97 | 90 | 175 |  |
| stage (%) |  |  |  | 0.241 |
| Stage I | 0 ( 0.0) | 0 ( 0.0) | 1 ( 0.6) |  |
| Stage II | 10 ( 10.4) | 4 ( 4.5) | 6 ( 3.4) |  |
| Stage III | 74 ( 77.1) | 73 ( 82.0) | 137 ( 78.7) |  |
| Stage IV | 12 ( 12.5) | 12 ( 13.5) | 30 ( 17.2) |  |
| grade (%) |  |  |  | 0.875 |
| G1 | 0 ( 0.0) | 0 ( 0.0) | 1 ( 0.6) |  |
| G2 | 12 ( 12.5) | 13 ( 14.4) | 17 ( 9.8) |  |
| G3 | 83 ( 86.5) | 76 ( 84.4) | 150 ( 86.2) |  |
| G4 | 0 ( 0.0) | 0 ( 0.0) | 1 ( 0.6) |  |
| GB | 0 ( 0.0) | 0 ( 0.0) | 1 ( 0.6) |  |
| GX | 1 ( 1.0) | 1 ( 1.1) | 4 ( 2.3) |  |
| Age = >60 (%) | 37 ( 38.1) | 43 ( 47.8) | 83 ( 47.4) | 0.281 |
| group (%) |  |  |  | <0.001 |

**Table S6.** The specific primers in this study.

| **Gene** | **Forward primer (5’ to 3’)** | **Reverse primer (5’ to 3’)** |
| --- | --- | --- |
| GAPDH | GTCTCCTCTGACTTCAACAGCG | ACCACCCTGTTGCTGTAGCCAA |
| PD-L1 | TGGCATTTGCTGAACGCATTT | TGCAGCCAGGTCTAATTGTTTT |
| PDCD1LG2 | ACCCTGGAATGCAACTTTGAC | AAGTGGCTCTTTCACGGTGTG |
| CTSS | TGTAGATGCGCGTCATCCTTC | CCAACCACAAGTACACCATGAT |
| GBP4 | ATGGGTGAGAGAACTCTTCACG | TGCGGTATAGCCCTACAATGG |
| TNF | CTCTTCTGCCTGCTGCACTTTG | ATGGGCTACAGGCTTGTCACTC |
| BST2 | CACACTGTGATGGCCCTAATG | GTCCGCGATTCTCACGCTT |
| IFITM1 | ACCAGTTCAAGAAGAGGGTGTT | CCAAGGTCCACCGTGATTAAC |
| PSMB8 | CCTTACCTGCTTGGCACCATGT | TTGGAGGCTGCCGACACTGAAA |

**Table S7.** Differential mutant genes between IRGs Cluster C1 and C2, C2 and C3.

| **Hugo_Symbol** | **P Value** | |
| --- | --- | --- |
|  | **C1 versus C2** | **C2 versus C3** |
| MYH7 | 0.01967 | 0.01717 |
| MYO3B | 0.01967 | 0.04421 |
| SLC6A20 | 0.01967 | 0.00390 |
| ZNF445 | 0.01967 | 0.04421 |
| BRCA2 | 0.02468 | 0.04569 |
| CDK12 | 0.04437 | 0.03567 |
| CCT8L2 | 0.04918 | 0.00635 |
| SPTB | 0.04918 | 0.01855 |
